# Immunotherapy Strategies for the Prevention and Treatment of Distinct Stages of Type 1 Diabetes: An Overview

**DOI:** 10.3390/ijms21062103

**Published:** 2020-03-19

**Authors:** Novella Rapini, Riccardo Schiaffini, Alessandra Fierabracci

**Affiliations:** 1 Diabetology Unit, Children’s Hospital Bambino Gesù, 00165 Rome, Italy; riccardo.schiaffini@opbg.net; 2 Infectivology and Clinical Trials Research Department, Children’s Hospital Bambino Gesù, 00165 Rome, Italy

**Keywords:** type 1 diabetes, high risk subjects, immunotherapy

## Abstract

Type 1 diabetes mellitus is a heterogeneous disorder characterized by destruction of pancreatic β cells, culminating in absolute insulin deficiency. The goals of Type 1 diabetes care, established by the Diabetes Control and Complications Trial (DCCT), are to achieve good glycemic control, to prevent hyperglycaemia (which is associated with long-term microvascular and macrovascular complications) and to avoid recurrent episodes of hypoglycaemia (which may have adverse effects on cognitive function). However, despite continuing optimization of insulin therapy regimes, the actual hormonal substitutive administration acts only to treat the symptoms without an effect on disease pathology and etiopathogenesis. In recent decades, a great deal of interest has been focused on prevention approaches in high-risk individuals, based on the hypothesis that a therapeutic intervention, if applied at the early stage of disease, might contribute to maintaining endogenous β cell function by preserving the residual β cell reservoir from autoimmune attack. This manuscript provides an overview of the most important immunotherapeutic interventions established so far for Type 1 diabetes treatment at different stages of disease that have reached an advanced stage of assessment.

## 1. Introduction

Type 1 diabetes (T1D) is a multifactorial autoimmune disease characterized by T cell-mediated destruction of insulin producing β cells of the pancreas. T1D accounts for 5–10% of diabetes cases worldwide, but its incidence has increased in recent years [1]. While T1D can be controlled by administration of exogenous insulin, there is no permanent cure, therefore a lifelong management is required. Both genetic and environmental factors are involved in the development of the disease. Major genetic risk factors are correlated with HLA (Human leukocyte antigen) genotype; moreover, outside the HLA region, the *INS* (insulin), *PTPN22* (protein tyrosine phosphatase N 22) [2], *CTLA4* (Cytotoxic T lymphocyte-associated protein 4), and *IL2RA* (IL-2 receptor) genes [3] exert the strongest effect on etiopathogenesis.

Autoimmune responses against β cells precede the clinical onset of T1D, when at least 80–90% of the β cell mass is lost [4]. Multiple immune cell types contribute to T1D pathogenesis, involving both innate and adaptive immune mechanisms, leading to expansion of autoreactive, antigen-specific T and B lymphocytes; these immunotypes are able to initiate islet inflammation to cause insulitis. Moreover, a reduction in T regulatory cell (Treg) numbers and functionality has been reported in T1D patients at onset [5,6,7].

The most frequent autoantibody specificities tested in the sera of T1D patients are directed to insulin (IAA), glutamic acid decarboxylase 65 (GADA), IA-2 antigen (IA-2A, insulinoma associated antigen 2) and zinc transporter 8 (ZnT8A) [8]. A “risk score” correlated to the probability of disease onset can be established based on the number and titer of circulating autoantibody specificities. If only one autoantibody is present, the risk of developing T1D is very small; however, if two or more than two autoantibody specificities are present, the probability of developing T1D is high, with a great variability in the progression rate. However, 80% of children with two autoantibody specificities are known to develop T1D within 15 years after seroconversion [9].

The most important knowledge of the natural history, epidemiology, heterogeneity and strategies to predict T1D onset has come from different cohort studies, such as DIPP (Type 1 Diabetes Prediction and Prevention) [10], BABYDIET study [11], TEDDY (The Environmental Determinants of Diabetes in the Young) [12], as well as TrialNet [13]. These studies were focused on screening and monitoring of first- and second-degree T1D relatives.

In particular, a recent review and update of the DIPP project and the TEDDY study highlighted that several investigators worldwide continue to improve our understanding of the etiopathogenesis of T1D, with the hope of ultimately preventing and reversing T1D. Indeed, over the last two decades, the DIPP project performed intensive longitudinal follow-up of newborns in the Finnish general population and more than 210,000 children have been screened for genetic susceptibility [14]. Simultaneously, the TEDDY Study has analyzed a large amount of data and blood samples from high-risk children followed during the first eight years of life, providing insights on T1D etiology and heterogeneity [15].

The natural history of T1D presents with several distinct stages, from the asymptomatic phase to clinical diagnosis, characterized by detection of specific islet-related autoantibodies and progressive destruction of β cells. These include a “pre-stage 1”, in which individuals carrying T1D susceptibility alleles have not yet developed islet autoantibodies. The development of two or more islet antibodies defines the “stage 1”, which can progress to “stage 2” when dysglycemia appears, and then progresses to symptomatic diabetes (stage 3) [16]. The decline in β cell number starts years before the symptoms of hyperglycaemia become evident. Moreover, we can identify the progression from “stage 1” to “stage 2” by an oral glucose tolerance test (OGTT) [17], or by an intravenous glucose tolerance test [18], which can detect a loss of first-phase insulin release due to decreasing β cell mass.

T1D develops with micro- and macrovascular complications that can lead to early deaths. As regards the long-term management of the disease, phenomena such as the ‘obesity paradox’, with an inverse correlation between Body Mass Index (BMI) and increased risk of mortality for cardiovascular failure in T1D, have been disputed in few epidemiological studies carried out over the past 10 years [19].

In the light of the foregoing, the development of appropriate preventive strategies is an urgent need in T1D management. A first approach is to reduce exposure to environmental factors that are triggers of disease in subjects harboring genetic markers (primary prevention). A second approach is to arrest the loss of β cells in the prediabetic stage or in autoantibody-positive subjects (secondary prevention). Although the disease has not been prevented, some studies have focused on the possibility of partially preserving residual β cell function by acting at the initial stage of disease onset (tertiary prevention). Moreover, different immunotherapy approaches have been developed in an attempt to modulate islet-specific immune pathways by targeting underlying defects within the different stages of the disease, in order to preserve β cell function and delay the clinical diagnosis without the toxicities produced by earlier immunosuppressive attempts.

Of great interest is the approach that the Juvenile Diabetes Research Foundation (JDRF) intends to apply in the near future in the field of primary, secondary and tertiary prevention. JDRF believes that, in the long-term, the most cost-effective approach is the prevention of pancreatic β cell autoimmunity, or primary prevention [16]. Nevertheless, secondary and tertiary prevention can be targeted to the presymptomatic and symptomatic stages of T1D by developing multi-interventions in order to halt the progressive loss of β cell function. These interventions are focused on targeting, in combination, inflammation of the pancreatic islets, β cell-specific autoimmunity, β cell survival, glucose intolerance and insulin resistance [20].

This review will discuss the most important immunotherapeutic interventions in T1D treatment, in the presymptomatic and symptomatic stages of T1D, so far established, and those at advanced stages of evaluation. We also emphasize the immunological effects of different treatments, either on T or B cells, or on antagonizing the effect of proinflammatory cytokines (Table 1, Table 2 and Table 3) [21,22,23,24,25,26,27,28,29,30,31,32,33,34,35,36,37,38,39,40,41].

## 2. Immunotherapeutic Approaches in the Presymptomatic Stage of T1D

The T1D prevention approach may represent a viable alternative to an actual cure by blocking the autoimmune response while β cell mass is still sufficient. For this reason, in the last few decades, prevention studies have started to focus on subjects at risk of developing the disease, in which the autoimmune process is ongoing but the β cell mass is still preserved. The most-used approach at this stage of T1D is as an antigen-specific therapy, based on insulin administration to establish tolerance. Overall, mechanistic long-term effects of antigen-tolerogenic treatments rely on effector T cell depletion with expansion of T regulatory cells (Treg) (Table 1).

The Diabetes Prevention Trial-Type 1 Diabetes (DPT-1) carried out two different studies to investigate whether parenteral or oral insulin could prevent or delay disease development in high-risk relatives of T1D patients (see above) [21] (Table 1). In the first study, participants received subcutaneous insulin injections twice daily (total dose 0.25 IU/Kg/day), plus, at baseline and at 12-month intervals, continuous intravenous infusions of insulin (for 4 consecutive days), at an initial dose of 0.015 U per kilogram per hour. Patients were followed up for 3.7 years. In the second study, participants received oral insulin capsules daily (7.5 mg/day) and were followed up for a mean duration period of 4.3 years; the primary endpoint was the diagnosis of diabetes. In both DPT-1 studies, insulin secretion before the diagnosis of diabetes was evaluated by the assessment of the C-peptide response during mixed-meal, oral glucose, and intravenous glucose-tolerance testing. There were no differences between groups in terms of the peak values or the areas under the curve for any applied test. The authors concluded that both subcutaneous and oral insulin administration did not prevent or delay T1D onset [21], although in a subgroup with anti-insulin autoantibodies persistently at high levels (> 80 nU/mL), a delay in the diagnosis of diabetes (as compared to the placebo group) has been demonstrated (Table 1). The Type 1 Diabetes TrialNet Study initiated a second prevention trial in relatives of T1D patients. Participants were assigned to receive, once daily, an insulin capsule (7.5 mg) or placebo. Participants were followed up for 2.7 years; T1D development did not significantly differ between the two groups [42].

The Type 1 Diabetes Prediction and Prevention study (DIPP) investigated whether nasal insulin could reduce the incidence of T1D [24]. The randomized double-blind trial recruited children at risk of developing the disease within the general population. Daily doses of intranasal insulin were administered; however, after 1.8 years of observation, no differences were found in the rate of progression to T1D [24] (Table 1).

The Intranasal Insulin Trial (INIT I) pilot study treated autoantibody-positive participants with intranasal insulin and showed that intranasal insulin did not prevent or accelerate T1D onset. In this trial, intranasal insulin administration correlated with increased antibody levels and decreased T-cell responses to insulin [37] (Table 1 and Table 3) [21,22,23,24,25,26,27,28,29,30,31,32,33,34,35,36,37,38,39,40,41]. The INIT II study is still ongoing and will expand the number of enrolled subjects. Thus, clinical trials evaluating insulin administration for disease prevention have demonstrated to date limited success in preventing the disease’s progression.

Another autoantigen used to interact with antigen-specific β cell autoimmunity is the Alhydrogen (aluminium hydroxide, Alum) formulate Glutamate Decarboxylase (GAD–Alum). In a recent paper [25], Elding Larsson H. et al. aimed, for the first time, to evaluate safety and efficacy of autoantigen-specific therapy with GAD–Alum (two doses of 20 mcg subcutaneously administered in nondiabetic children with multiple islet autoantibodies). The study (named DiAPREV-IT) was a randomized clinical trial versus placebo and did not demonstrate the efficacy of GAD–Alum autoantigen therapy in preventing progression to T1D. Treatment produced an increase in Treg cells, which are supposed to down-regulate GAD65-specific autoreactive T cells (reviewed (rev) in [25]) (Table 1 and Table 3) [21,22,23,24,25,26,27,28,29,30,31,32,33,34,35,36,37,38,39,40,41].

Recently, the use of immunosuppressive drugs has also been proposed as a possible approach to decelerate the evolution of the disease. As regards this, a multicenter, randomized, placebo-controlled clinical trial based on Abatacept (CTLA-4 Ig) administration in subjects from the Trialnet study at risk to develop T1D is ongoing (NCT01773707). Abatacept is an immunomodulatory molecule-blocking T cell co-stimulation modulator, having impact on the rate of reduction of β cell function (Table 1). The primary endpoint will be to determine whether this intervention will prevent or delay T1D in relatives with two or more islet autoantibodies and normal glucose tolerance. The trial is based on the finding that Abatacept is supposed to delay the loss of insulin secretion in recent-onset diabetes.

Finally, the Type 1 Diabetes TrialNet Study Group tested, in a recent trial [26], whether teplizumab treatment would prevent or delay the onset of clinical T1D in high-risk subjects. Teplizumab is a humanized FcR non-binding monoclonal anti-CD3 antibody targeting CD8 lymphocytes, important effectors of β cell injury in T1D etiopathogenesis (Table 1 and Table 3) [21,22,23,24,25,26,27,28,29,30,31,32,33,34,35,36,37,38,39,40,41]. Clinical efficacy is related to the increased frequency in this subset of markers of T cell exhaustion, with increased expression of T cell immunoreceptors with Ig and ITIM domains (TIGIT), killer cell lectin-like receptor G1 (KLRG1) and eomesodomain (EOMES) [43]. Patients were randomized (1:1) to teplizumab or placebo. In the treatment group, participants received a 14-day outpatient course of teplizumab to be administered at a growing dose of 51 to 826 μg per square meter of body surface area. The authors demonstrated that, although the trial had limitations and the cohort was small, a 2-week course of treatment with teplizumab delayed the diagnosis of clinical T1D in high-risk participants with a reduction in the annualized diagnosis of the disorder.

## 3. Studies in the Symptomatic Phase of T1D

Several immunomodulatory and immunosuppressive agents have been tested, alone or in combination, in an attempt to block the immune-mediated destruction of β cells that occurs in T1D. The majority of the studies have been performed in patients at disease onset, at which time most of the β cell mass has already been lost. However, in this stage of the disease, patient recruitment is easier and more justified, and the major outcome is preservation of the residual β cell function.

This approach has produced limited long-term success, principally due to significant immunosuppressive side effects that precluded therapeutic outcomes.

Anti-CD3 antibody was administered to recent-onset T1D patients. Unfortunately, these drugs showed transient efficacy in new-onset patients who remained insulin-dependent after the therapy [32,33,44]. A single course of anti-CD3 monoclonal antibody hOKT3gamma1 (Ala-Ala) (teplizumab) resulted in improvement in C-peptide responses and clinical parameters for at least 2 years after T1D onset [38]. The anti-CD3 antibody teplizumab did not meet primary endpoints for insulin usage after 1 year (0.5 unit/kg/day) with HbA1c < 6.5%) [44]. C-peptide release was preserved by 30%. Significant efficacy of the treatment was observed after 2-year follow-up with good residual insulin secretion. Side effects were not increased compared to controls [45]. Anti-CD3 antibody otelixizumab revealed a narrow therapeutic window. At a low dose, there was no preservation of β cells [46]. This was indeed observed at 18 months, but with significant adverse effects [47]. Otelixizumab is a chimeric monoclonal antibody that targets the CD3/T-cell receptor, which is genetically modified by removing the glycosylation site in the Fc domain, thus affecting binding of complement or Fc receptors. This reduces secondary reactions due to cytokine release. Otelixizumab downregulates pathogenic T cells and upregulates Treg cells, thus halting the autoimmune process in T1D (rev in [46]) (Table 3).

Only one trial has focused on β cell function in T1D after treatment with Rituximab, which produces B cell depletion [27] (Table 2 and Table 3). Although T1D is a T-cell mediated autoimmune disorder, B lymphocytes play a pathogenetic role by acting as antigen-presenting cells (APC) and modulating the islet environment. In recent-onset T1D patients, administration of Rituximab reduced HbA1c levels and exogenous insulin demand due to the preservation of C-peptide levels over 1 year [47]. Interestingly, after 2 years’ follow up, Rituximab delayed the decrease in C-peptide levels but did not influence insulin dose [28], suggesting B cell deletion is not sufficient to restore β cell tolerance.

CTLA4-Ig (Abatacept) has been administered in recent-onset T1D; in a trial by Orban and colleagues [29], Abatacept was administered on days 1, 14, 28, and then every 28 days through a 30 min intravenous infusion at a dose of 10 mg/kg. Abatacept was more efficient compared to placebo in preserving the β cell mass, as evidenced by stimulated C-peptide secretion. However, the effect diminished with time; therefore, further investigation will be necessary in order to unravel whether the beneficial effect persists after cessation of infusions.

Indeed, Abatacept is a potential candidate to be used in tertiary prevention trials, and is a candidate useful component of combination therapies in recent-onset T1D patients [29].

Interventions have also focused on inflammation at onset of disease. As regards this, etanercept, a TNF-α inhibitor, has been used to treat recent onset T1D patients, and induced an improvement of HbA1c and C-peptide levels [30]. Etanercept is a recombinant soluble TNF-α receptor fusion protein, which acts by blocking the biological activity of the inflammatory cytokine TNF-α, which potentiates β cell destruction in concert with other cytokines such as interleukin-1β and interferon-γ. As shown in NOD (non-obese diabetic) mice TNF-α mRNA is produced by CD4 T-cells within insulitis, during the development of diabetes (rev in [30]) (Table 3).

IL-1 has also been investigated as a potential target for T1D prevention. Nevertheless, studies employing Anakinra, an IL-1 receptor antagonist (IL-1ra) failed to show an improvement of β cell preservation [31,39] (Table 2 and Table 3).

Another therapeutic approach is to reestablish antigen-specific tolerance by in vivo presentation of β cell antigens, resulting in inactivation of autoreactive diabetogenic T cells or induction of antigen-specific immunoregulatory populations, in order to prevent autoimmune processes.

A recent study evaluated the efficacy of intralymphatic injection of GAD65 in an aluminum hydroxide-formulated vaccine (GAD-alum) for the treatment of recent onset T1D patients. This approach showed great promise in phase 2 trials [48] based on results for C-peptide levels, but failed in phase 3 [49]. The same vaccine was administered in children at risk to develop T1D, who were followed up for 5 years (DIAPREV-IT). The study failed to show any effect on the progression to T1D [25]. Lower doses of GAD-alum administered into lymphonodes induced higher GADA levels than subcutaneous injections with reduced IgG1 GADA and increased IgG2, IgG3, and IgG4 subclasses. GAD65 stimulation induced activated CD4 T cells and a Th2 cytokine profile (Table 3).

Cell-based therapies are mainly focused on Treg use. Based on the evidence that Treg cells are defective in T1D patients, restoring their function by transferring ex vivo-expanded Treg cells back into the donor may reverse the autoimmune process and preserve the residual β cell mass. Phase I trials using this approach showed an increase in C-peptide levels and a reduction of exogenous insulin requirement [50]. In a study conducted by Bluestone and colleagues, no decline in C-peptide levels and no worsening in HbA1c over 1 year post-transfer was observed [34].

Taken together, these early data suggest that Treg therapy may be beneficial for preserving β-cell mass and possibly reversing T1D. A more recent Phase III clinical trial explored the use of antithymocyte globulin (ATG) + granulocyte colony-stimulating factor (G-CSF) in T1D patients as a combination therapy. This found no differences in C-peptide levels with a 2-year follow-up but Treg cells were elevated after 6, 12 and 18 months [35]. Treatments reduced circulating CD4:CD8 ratio, while increasing that of Treg cells: conventional CD4 T-cells, memory CD45RO+ Treg cells, and TIGIT (T cell Immunoreceptor with Ig and ITIM domains) + Treg cells, known to suppress T helper 1 and 17 cells. ATG with or without G-CSF resulted in persistent upregulation of PD-1 (programmed cell death 1) molecules in total CD4+ T cells [35] (Table 3).

In the light of the foregoing, these results suggest that ATG/G-CSF therapy leads to a more slow autoimmune destruction of β cell mass in T1D patients.

## 4. Trials Ongoing

Several clinical trials are still ongoing through stage 2 and stage 3 of T1D evaluation.

In the Fr1da study [23] participants have been identified as at risk to develop T1D by population-based screening. Fr1da participants are randomized to receive oral insulin capsules or placebo. The study is still ongoing, and outcomes will be evaluated based on the rate of progression of dysglycemia and the change from baseline in CD4+ and regulatory T cells [23].

Moreover, a trial using Alum-formulated GAD (Diamyd), in combination with high-dose Vitamin D3 in children at risk to develop T1D is ongoing (NCT02387164).

A two-arm, multicenter, 1:1 randomized, placebo-controlled clinical trial will investigate the effects of CTLA-4Ig (Abatacept) on prevention of abnormal glucose tolerance (AGT) and diabetes development in relatives at risk for T1D. The primary objective is to determine whether intervention with Abatacept will prevent or delay the development of AGT in at-risk autoantibody-positive non-diabetic relatives of T1D patients. Secondary outcomes include the effect of Abatacept on the incidence of T1D, analyses of C-peptide levels’ safety and tolerability, and mechanistic outcomes (NCT01773707) (Table 1).

Another ongoing interventional clinical trial will test the efficacy and safety of the sequential administration of Rituximab followed by Abatacept in individuals at risk to develop T1D (NCT03929601).

Finally, a pilot study (POSEIDON) will investigate the safety and efficacy of a regimen that combines Omega-3 Fatty Acids and Vitamin D in subjects at T1D onset (NCT03406897). Vitamin D is known to have effects on the generation and function of Treg cells [40] (Table 3). Omega-3 Fatty Acids have effects on several immunotypes including macrophages, neutrophils, eosinophils, basophils, dendritic, NK and mast cells, and B and T lymphocytes; further, they are known to increase Treg cell differentiation [41] (Table 3).

## 5. Conclusions

Although the pathogenesis of T1D remains still to be unraveled, our knowledge of the natural history of the disease has increased over the years. In particular, we can now identify subjects at risk to develop the disease before diagnosis, offering the possibility to modulate this preclinical stage with a variety of therapeutic approaches both for primary and secondary prevention, aiming to preserve β cell mass.

Experimental animal models have already demonstrated that mucosal immune tolerance, induced by administration of autoantigens, can prevent the onset of autoimmune disease [51].

A variety of immunotherapies for treating or preventing T1D have been unraveled in the past decade; however, completed studies so far have been unsuccessful in significantly preserving β cell mass from the autoimmune attack. Overall, as shown in Table 2, different immunotherapies have aimed to target pathogenic immunological mechanisms caused by pathogenic autoreactive T cells [19,20,21,22], by blocking costimulation [27], inducing exhaustion of expanded CD8+ T cells [37] or blocking the effect of proinflammatory cytokines (i.e., TNFα, IL-1β) [30,31,39]. Treatments can also induce Treg cell generation, stimulate their function, and increase the Treg/Teff ratio [35]. Specific therapies (i.e., Rituximab) can also influence antigen presentation and autoantibody production by inducing B lymphocyte depletion [27] or by having effects on other immunotypes (specifically Omega-3 fatty acids), including macrophages, dendritic cells and NK cells [40]. Indeed, no primary or secondary prevention trial was able at the present stage of evaluation to prevent the development of clinical diseases in at risk subjects. More clinical and molecular studies are needed to better understand the inflammatory process that occurs in the islets of Langerhans at different stages of T1D development, to improve therapeutic approaches targeted to prevent and reverse β cell mass destruction. The most practical approach is to halt evolution of β cell dysfunction by intervening near the disease onset.

More individual and combination immunotherapies may potentially be experimented with in the future to ameliorate glycemic control and preserve β cell function, thus contributing to an effective prevention approach for the disease. Future studies should be based on improved designs in the preclinical and clinical settings. Future, more refined, approaches should specifically modulate islet-specific mechanisms underlying tolerance, avoiding toxic effects of earlier broad immunosuppressive drugs. Targeted therapies should therefore be based on a deeper understanding of the interactions between genetic background, environmental factors, triggers of disease and immunological mechanisms.

## Figures and Tables

**Table 1:** Trials at type 1 diabetes (T1D) stage 1.

Prevention Trial.	Drug	Study	Number of Enrolled Patients	Outcome	Status	Ref.
NCT00004984 (DPT-1)	Parental or oral insulin	Phase 3, Secondary prevention	372, oral insulin 339, parenteral insulin	No protective effect	Completed	[21,22]
NCT00336674 (INIT-II)	Intranasal insulin	Phase 2, Secondary prevention	/	/	Active not recruiting	Ongoing
NCT02620072 (Fr1da)	Oral insulin	Phase 2, Secondary prevention	/	/	Recruiting	[23]
NCT00223613 (DIPP)	Intranasal insulin	Phase 3, Secondary prevention	264	No protective effect	Completed	[24]
NCT01122446 (DIAPREV-IT)	Diamyd (GAD-Alum)	Phase 2, Secondary prevention,	25	No protective effect	Completed	[25]
NCT01773707 (NIDDK)	Abatacept (CTLA4-Ig)	Phase 2, Secondary prevention	/	/	Recruiting	Ongoing
NCT02387164 (DIAPREV-IT2)	Diamyd (GAD-Alum) + Vitamin D	Phase 2, Secondary prevention	/	/	Active not recruiting	Ongoing
NCT01030861	Teplizumab	Phase 2, Secondary prevention	44	delayed progression	Completed	[26]
NCT03929601	Rituximab	Phase 2, Secondary prevention	Estimated 36		Completed	

**Table 2:** Prevention trials at T1D onset.

Prevention Trial.	Drug	Study	Number of Enrolled Patients	Outcome	Status	Ref
NCT00279305	Rituximab	Phase 2, T1D onset	57	delayed fall in C-peptide	Completed	[27,28]
NCT00505375	Abatacept (CTLA4-Ig)	Phase 2, T1D onset	77	delayed fall in C-peptide	Completed	[29]
NCT00730392	Etanercept (TNF-α inhibitor)	Phase 1-2, T1D onset	9	delayed fall in C-peptide	Completed	[30]
NCT00645840	IL-1 beta (anakinra)	Phase 1-2, T1D onset	46	No effect	Completed	[31]
NCT03406897 (POSEIDON)	Omega-3 fatty acids and Vitamin D	Phase 1-2 (Pilot study), T1D onset	/	/	Recruiting	ongoing
NCT00129259	Teplizumab	Phase 2, T1D onset	52	delayed fall in C-peptide	Completed	[32,33]
NCT01210664	Polyclonal Treg	Phase 1, T1D onset	14	delayed fall in C-peptide	Completed	[34]
NCT01106157	Antithymocyte globulin (ATG) and G-CSF	Phase 1 -2, T1D onset	17	delayed fall in C-peptide	Completed	[35,36]

**Table 3:** Effects of different immunotherapies on immune cells.

Treatment	Immune Cell Effect	Ref.
insulin	reduced T cell responses; increased antibodies	[21,22,23,24,25,26,27,28,29,30,31,32,33,34,35,36,37]
GAD-Alum	increased Treg	[25]
Abatacept (CTLA4-Ig)	reduced T cell costimulation	[29]
Teplizumab (anti-CD3)	increased CD8 T cells with markers of T cell exhaustion	[26]
Otelixizumab (anti-CD3)	increased Treg; downregulation of pathogenic T cells	[38]
Rituximab (anti-CD20)	B lymphocytes depletion	[27]
Etanercept	blocking TNF-α	[30]
Anakinra (IL-1ra)	antagonism on IL-1 receptor	[31,39]
ATG + G-CSF	reduced CD4:CD8 T cell ratio; increased Treg/conventional CD4 T cell ratio; increased CD45RO+ Treg; increased TIGIT+ Treg	[35]
Vitamin D	influence on Treg generation and function	[40]
Omega-3 fatty acids	effect on macrophages, neutrophils, eosinophils, basophils, dendritic, NK and mast cells, B and T lymphocytes; increase of Treg differentiation	[41]

## Abbreviations

ATG	Antithymocyte globulin
BMI	Body Mass Index
CTLA-4 Ig	Cytotoxic T lymphocyte-associated antigen 4-immunoglobulin (CTLA4-Ig)
DiAPREV-IT	The Diabetes Prevention–Immune Tolerance (DiAPREV-IT) studies
DIPP Type 1	Diabetes Prediction and Prevention
EOMES	eomesodermin
Fr1da	Fr1da-Insulin-Intervention study
GADA	Glutamic Acid Decarboxylase Autoantibodies
GAD-Alum	Glutamic Acid Decarboxylase-alum
G-CSF	granulocyte colony-stimulating factor
HbA1c	Glycated hemoglobin
IA-2A	Autoantibodies against protein tyrosine phosphatase insulinoma associated antigen 2
IAA	antibodies against insulin
IL2RA	IL-2 receptor
INS	Insulin (gene)
IL	Interleukin
JDRF	Juvenile Diabetes Research Foundation
KLRG1	killer cell lectin-like receptor G1
PD1	programmed cell death 1
PTPN22	Protein tyrosine phosphatase N22
rev	reviewed
T1D	Type 1 diabetes
TIGIT	T cell Immunoreceptor with Ig and ITIM domains
TNF	Tumor necrosis factor
Treg	Regulatory T cells
ZnT8A	zinc transporter 8 autoantibodies
